# Carbon contracts-for-difference: How to de-risk innovative investments for a low-carbon industry?

**DOI:** 10.1016/j.isci.2022.104700

**Published:** 2022-07-01

**Authors:** Jörn C. Richstein, Karsten Neuhoff

**Affiliations:** 1 Climate Policy Department, German Institute for Economic Research (DIW Berlin), 10117 Berlin, Germany; 2 Chair of Climate and Energy Policy, Technische Universität Berlin, 10623 Berlin, Germany

**Keywords:** Energy flexibility, Energy Modelling, Energy policy, Energy resources

## Abstract

The shift to climate neutrality requires new process technologies for energy-intensive industries, such as steel, chemicals, or cement. A variety of technology options exist – but they face the challenges of (i) first-of-kind costs, (ii) higher operation and investment costs, and (iii) insufficient and uncertain carbon prices, which partly stem from political uncertainty. Existing innovation policy instruments and carbon policies such as price floors can only partly address these challenges. Project-based carbon contracts-for-difference (CCfDs) guarantee investors a fixed carbon price over the contract duration, thus de-risking such investments from political and market uncertainty, and allowing governments to set carbon prices above current levels. Here we show for a case study in the steel sector that carbon mitigation costs can be reduced by up to 27% and that owing to high incremental operation costs, price floors of 79% of the CCfD price would be needed for projects to achieve bankability.

## Highlights

• Low-carbon industry faces higher costs but low and uncertain carbon prices
• Project-based carbon contracts-for-difference (CCfD) secure revenue from carbon savings
• When incremental OPEX are high, high price floors needed to enable bankability
• CCfDs lower carbon mitigation costs for steel by up to 27% compared to no policy

## Graphical abstract

**Figure:**
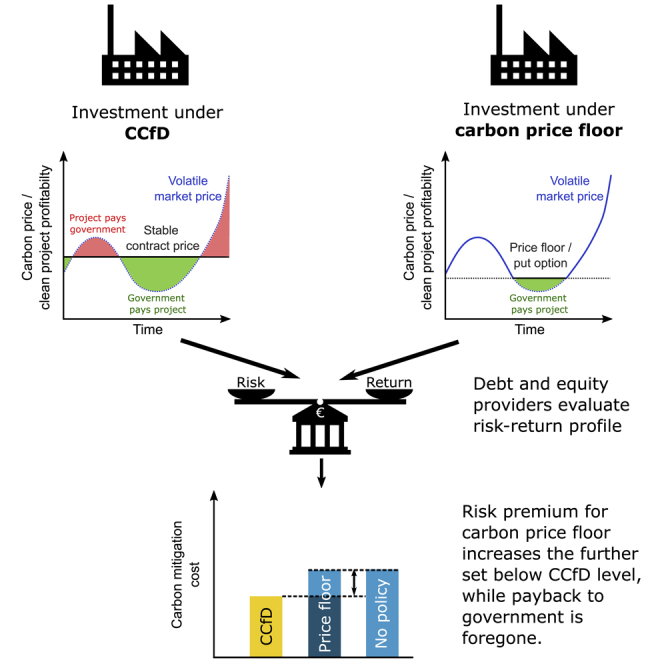

## Introduction

Energy-intensive industries, including iron and steel, cement, chemicals and petrochemicals, pulp and paper, and aluminium, account for 25–33% of carbon emissions and 38% of total final energy use worldwide (Allwood et al., 2010; IEA, 2017). These basic material-producing industries are mostly considered hard to decarbonize (Chiappinelli et al., 2021). Several innovative technologies already exist to significantly reduce emissions in these sectors (Bataille et al., 2018; IEA, 2017), compatible with the net-zero pathways announced worldwide, including by the European Union. Initiating the decarbonization with early deployment of these technologies is economically efficient owing to the longevity of assets (Vogt-Schilb et al., 2018). Reducing carbon emissions requires actions both from governments and private parties. Both carbon pricing, as well as further policies are in principle necessary to direct innovation and research in the direction of clean technologies (Acemoglu et al., 2012). In the past, significant levels of public and private R&D funding have taken place in the energy-intensive industries and led to a series of pilot projects (for example in Europe in the steel sector funded by the Horizon 2020 program, as well as the NER 300 fund (Kushnir et al., 2020)), bringing technologies (close) to large-scale maturity. In the long-run, sufficient carbon pricing (including carbon leakage protection) promises to provide a framework to ensure private investments in clean processes (Bataille et al., 2018), potentially complemented or substituted by phasing-out policies at later stages of the diffusion process, such as product carbon requirements (Gerres et al., 2021). However, currently private investments in these technologies face the challenges of (i) first-of-kind costs (ii) higher operation and investment costs than conventional carbon-intensive processes and (iii) insufficient and uncertain carbon prices, which partly stem from political uncertainty, and which, owing to incomplete risk markets (Greenwald and Stiglitz, 1986), can usually not be hedged over sufficient time horizons (Newbery et al., 2019).

Thus, currently, many innovative, but technically mature, basic material production processes face a valley of death (Nemet et al., 2018), where the clear roles of government (providing basic R&D funding), and private industries (diffusing mature technologies under stable government frameworks, such as carbon prices) intersect, and policy support may be needed for the early deployment and commercialization of innovative production processes (cf. further policies to incentivize the efficient use and recycling of materials in the discussion).

The discussion paper predecessor (Richstein, 2017) to this article proposed project-based carbon contracts-for-difference (CCfDs) between governments and innovative low-carbon projects in energy-intensive industries to overcome these hurdles. CCfDs guarantee investors stable revenue from carbon savings. When carbon prices in markets or reflected in materials are below the agreed contract price (i.e. the strike price in the contract), the government pays out the difference for each ton of reduced emissions compared to the conventional technology. In exchange, when the carbon price exceeds the contract price the project pays back this difference to the government. As many industrial innovative projects need carbon prices exceeding current levels, eligibility is proposed to be limited to technologies in line with net-zero pathways of governments, and as a result, contracts should be directly linked to a project. This concept is in the process of implementation in Germany (BMU, 2021), and is under consideration by the European Union and several member states. Similarly, the Netherlands has already implemented the SDE++ program, which is comparable to CCfDs, but is implemented as a one-sided project-specific put option (i.e. a project-specific floor price, which ensures projects against low prices, but does not include a pay-back to the government in case of high carbon prices), and targeted more broadly towards several economic sectors.

The benefits of government guarantees for carbon prices have been discussed qualitatively by Helm & Hepburn (2007), who propose carbon contracts to address the credibility problem governments face. Government-issued carbon contracts or put-options have been discussed as means to show commitment to the carbon market, and stabilize carbon prices (Chiappinelli and Neuhoff, 2020; Ismer and Neuhoff, 2009; Zachmann, 2013). More generally, carbon price guarantees have also been proposed qualitatively as a way of supporting deep decarbonization technologies such as carbon capture and storage (Groenenberg and de Coninck, 2008; von Stechow et al., 2011). Similarly, policies for renewable energies have demonstrated that de-risking such investments via revenue-stabilizing policy frameworks can lead to lower carbon mitigation costs, via better financing conditions (Egli et al., 2018, May and Neuhoff, 2021). Design options of CCfDs have been discussed in several policy reports (Gerres and Linares, 2020 n.d.; Hauser et al., 2021; Lösch et al., 2021; Neuhoff et al., 2019; Sartor and Bataille, 2019), including in the form of a project-specific put option (McWilliams and Zachmann, 2021), and several research articles reference CCfDs as a useful part of a policy toolbox (Chiappinelli et al., 2021; Löfgren and Rootzén, 2021; Muslemani et al., 2021; Sutherland, 2020). Vogl. et al. (2021a) critically compare CCfDs with regulated demand-side creation in the context of the steel sector and find that CCfD are the superior policy option for early commercialization, as the creation of mandated green steel markets, results in additional risks, or cannot account for the heterogeneity of products (while it may be more useful for creating international demand for green steel). This article complements the previous literature by being the first to quantify the impact of CCfDs (or their absence) on investment choices and financing conditions in energy-intensive industries, and the potential benefits in terms of abatement cost savings. It more generally demonstrates the importance of derisking policies in the presence of both incremental investment and operation costs.

We develop a general analytic framework to determine necessary expected carbon price levels for low-carbon investments which are bankable (i.e. can be financed with a positive debt share), and quantify and extend the implications using a numerical simulation based on a Conditional-Value-at-Risk (CVaR) approach. We use common project-finance assumptions to model the implications of financing costs of different policy choices. For a case study of low-carbon steel (iron and steel is the single largest emitting industrial sector at 7% of total greenhouse gas emissions (IEA, 2020)), we find in our reference scenario that CCfDs reduce carbon mitigation costs by up to 27%, as compared to an absence of any derisking policy, and by 14.1% as compared to a price floor set at a level to just enable the project to take on debt capital. For project-specific put options and price floors, we find that their strike price needs to be similar to the strike price of a CCfD if operational costs of the low-carbon technology are high. Put options create, therefore, similar costs to governments if carbon prices turn out to be low, but do not offer the benefit of paybacks for governments if carbon prices are high. Price floors, therefore, would need to be set at very high levels to facilitate project realization, potentially exceeding the level that would be politically feasible in many jurisdictions.

## Results

### Financing and bankability of projects with incremental OPEX

To analyze the effect CCfDs have on investment decisions in low-carbon technologies with higher operational expenditures (OPEX) and capital expenditures (CAPEX) than conventional technologies, we develop an analytical framework for estimating financing structures and costs of bankable projects, based on project finance methodologies and practices (Gatti, 2008). Similar risk-return trade-offs hold for projects backed by corporate finance (also cf. STAR Methods). In the analytical analysis, we determine what financing conditions (in terms of debt-equity shares) are feasible under the uncertain product and carbon prices in the presence (or absence) of CCfDs and carbon price floors. We further analyze the level of expected carbon break-even prices that are necessary to enable investments in a low-carbon technology, when the conventional technology remains price setting (as we expect for global commodity markets in the coming years). The model captures three requirements that are necessary for financing an investment project:

• Operating condition: Firms will only operate the process if product revenues including savings on carbon costs are sufficient to cover operational expenditures. This is especially relevant as many low-carbon technologies in energy-intensive industries have higher operational costs than conventional technologies.
• Debt-servicing condition: The providers of debt and bonds require a very high likelihood of pay-back of debt and bonds (Gatti, 2008) – in exchange for their provision of low-cost capital. Only the share of revenues exceeding operating costs at such a high likelihood can be used to secure debt.
• Break-even condition: project owners will only invest in a project if in expectation the project revenues cover all operational and investment costs.

Several insights can be derived from the theoretic model: (1) secured carbon prices and especially CCfDs help in achieving bankability by securing revenue streams so that increasing the share of debt finance that is possible, (2) As debt is less expensive than equity, the enhanced bankability and improved operational conditions reduce the carbon price at which a project is viable with a CCfD compared to the carbon price floors that would be necessary for viability. (3) The higher the incremental operational costs are compared to the (debt annualized) investment cost, the more carbon price floors (or the realization of carbon contracts as put options) need to approach CCfD levels to enable bankability of projects.

We quantify the effect using a case study on a near climate-neutral steelmaking process, namely the hydrogen-based direct reduced iron process (Vogl et al., 2018) (H2-DRI) route. The H2-DRI route is the prospective medium-term low-carbon solution favored by the industry to replace the dominant conventional coal-based blast-furnace basic oxygen furnace (BF-BOF) route, as reflected in worldwide announced low-carbon steel projects (Vogl et al., 2021b). We parameterize the analytical model for the H2-DRI and BF-BOF routes (cf. STAR methods) for a European full-scale steel plant to start production in the time frame of 2025 to 2030. We will first discuss if the analytic model is a good fit for past operating margins in the steel industry. Then, for investments in low-carbon technology, we discuss the cases for carbon prices (and price floors) in which the debt-service condition (i.e. debt levels above zero) and the operational condition are always achieved; in the next section, this will be extended to cases where projects are purely equity funded with increasing risk premiums for higher levels of carbon price uncertainty.

**Figure 1:**
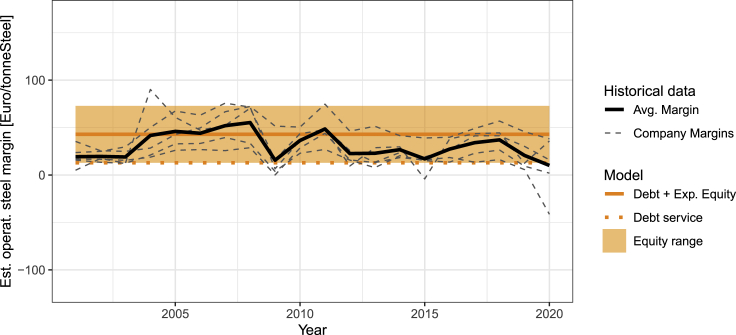
Historical data and modeled steel margins for conventional process Related to the STAR Methods and Table S1.

Figure 1 shows historical estimated operating margins (for crude steel production for 5 major steel companies active in the European market, as well as the sector average, expressed in Euro per tonne of steel produced (based on EBITDA margins and crude steel variable costs, cf. STAR methods). This margin represents the share of revenue available to serve creditors and equity stakeholders after operational costs have been accounted for. We compare this to the margins for the conventional BF-BOF process, which we calculate from the debt-equity shares and costs of debt and equity in the steel sector (Damodaran, 2020), if debt-servicing and break-even conditions hold. In this case, steel margins should in nearly all cases suffice to serve creditors. This holds for the historical average industry margin, which is above the level required for debt service in all years except 2020. Secondly, the margins above the level of debt service should be in a range consistent with equity expectations of returns. This also holds, although over the 20-year average margins have been slightly below the current estimated expected returns on equity (Damodaran, 2020).

**Figure 2:**
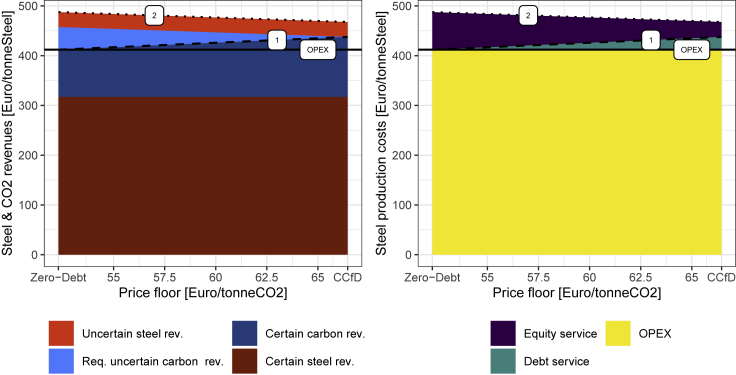
Certain and uncertain revenue streams and financing structures The debt-servicing condition (dashed line 1) and break-even condition (dotted line 2) determine the required carbon revenues for investments to take place.

Figure 2 shows how an increasing carbon price floor (the x-axis) increases the share of certain revenues (left panel), and therefore allows for increasing the use of debt in the financing structure (right panel). On the right side of each graph, no uncertain carbon price revenue is required to attract equity investors. This corresponds to a competitively priced CCfD).

A large share of costs is equally born by all global steel producers (such as iron ore prices). Hence, any cost changes of these costs are passed to product prices albeit sometimes with delays. We, therefore, abstract from these variations and consider the corresponding costs and revenue secure (included in certain steel revenue and OPEX costs, respectively). Scarcity prices on local and global steel production capacity do result in additional revenues, which are, however, uncertain for any steel production process (light red area in the left panel).

Carbon savings translate into revenues if carbon costs are reflected in steel prices set by conventional production processes, or if clean processes are supported by free allocation. These savings are subject to uncertainty about future carbon price developments. This uncertainty is considered to be very high, unless a carbon price floor can guarantee a minimum of secure revenues or CCfDs secure the full savings. The required carbon revenues from carbon prices exceeding an assumed carbon price floor are calculated by determining the project break-even condition (dotted line 2 in Figure 2).

The right panel of Figure 2 illustrates how reducing carbon price uncertainty (owing to an increased carbon price floor) reduces the expected carbon price required for the project to break even. It increases the share of the revenue streams that are certain (dashed line 1 in Figure 2). This revenue stream is primarily used to pay for OPEX (labor costs, iron ore, and so forth). Any remaining part of the certain revenue stream can be used to raise and serve debt (green area). Debt allows for financing at low-interest rates (at around 3% in the steel sector (Damodaran, 2020)). With an increasing carbon price floor, more certain revenue is available to serve debt. More debt can be raised to finance the investment. This reduces the amount of equity that is required. As equity capital (purple area, at around 10% (Damodaran, 2020)) requires higher rates of return, overall costs decline if equity financing can be partially substituted by debt financing.

The model is calibrated to values on costs and margins of the conventional BF-BOF process, as well as costs of the new process (cf. previous section and STAR methods) to determine the carbon prices required for the investment to take place (and resulting carbon break-even prices).

This analytic model is only valid for a range of carbon price floors. For carbon price floors below 52 Euro/tonneCO_2_, revenues become insufficient to cover any level of debt. All investment needs to be funded through equity (left side of x-axis). In the next section, we will model the implications for the break-even condition using a CVaR approach. For carbon price floors exceeding 66 Euro/tonneCO_2_, the debt share is not further increased as other uncertainties linked to the volatility of scarcity mark-ups in steel markets constrain further leverage. In this case, additional carbon revenue from potential prices exceeding 66 Euro/t are not necessary for the financial viability of an investment choice. With CCfDs they would be returned to society, while in the case of put-options or hypothetical price floors at such a level they would provide additional profits for investors.

**Figure 3:**
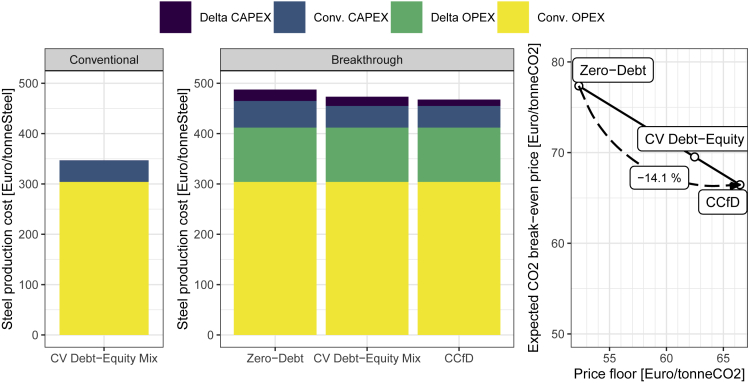
Steel production costs of the conventional and breakthrough technology under varying financing structures We show the points of zero-debt, the conventional debt-equity mix (CV Debt-Equity), and a CCfD debt-equity mix, as well as the resulting carbon break-even prices.

To illustrate, which elements drive break-even carbon prices, Figure 3 displays the steel production cost of the conventional BF-BOF route (left panel), and for the new H2-DRI route under three policy choices and resulting financing structures. The production costs are further differentiated in the OPEX and CAPEX levels of the conventional technology and of the H2 DRI route (as in the analytical model, cf. STAR methods).

In the CCfD case, the incremental investment cost as compared to the conventional route can be fully debt-financed as carbon price revenues are secure, whereas the remaining conventional CAPEX are financed at the conventional debt-equity structure, as conventional steel price risks remain to be covered. This results in a break-even carbon price based on the incremental CAPEX (the incremental investment ΔI annualized with debt annuity factor $a_{D}$) and incremental OPEX ($\text{Δ}c_{v}$) only, divided by the achieved emissions reductions (Δε):

$$p_{CCfD}=p_{minCO2}=\frac{a_{D}\cdot \text{Δ}I\;+\text{Δ}c_{v}}{\text{Δ}\varepsilon }\;$$

With a price floor or put option set at the level to secure the operation of the project, the entire project is equity-financed which leads to higher financing costs as compared to conventional steel projects. Interestingly, considering the interaction of different uncertainties shows that projects can be financed even if price floor levels are below the carbon price level required to cover incremental operational costs (cf. STAR methods). This is feasible because lower debt shares than under conventional steel projects are utilized, while the secure basis of steel product margins remains to ensure positive operational margins overall. However, owing to the significant share of incremental operational costs, the price floor that allows for positive debt levels is only around 21% below the break-even CCfD price. This small reduction of the price floor already translates to an increase in the expected carbon price that has to reach for the project to break-even price by 16.4% to 77 Euro/tonneCO_2_.

In the supplemental information, we assess how alternative cost assumptions, especially OPEX cost, the effect break-even prices (cf. Figures S2–S5). We find that especially electricity costs are the main price driver of carbon mitigation costs and CCfD contract prices.

### Equity premiums for projects without debt share

Proxying financing costs using debt-equity shares is not viable if the carbon prices floor declines below the level at which zero-debt is reached. To model such scenarios, we apply a Conditional-Value-At-Risk (CVaR) Monte-Carlo simulation, so as to capture the equity premiums for investments in low-carbon projects, including in those cases where projects are unbankable (cf. STAR methods). As before the resulting break-even, carbon price is endogenously determined in the model framework, as is the resulting distribution of carbon prices, for each exogenously given carbon price floor scenario. We calibrate the model to empirical equity costs in the steel sector. However, as the CVaR model offers two parameters to match empirical data, several parameter combinations provide a good fit. We choose a central scenario as the main one to analyze, which at the same time has a good fit with the analytic model.

**Figure 4:**
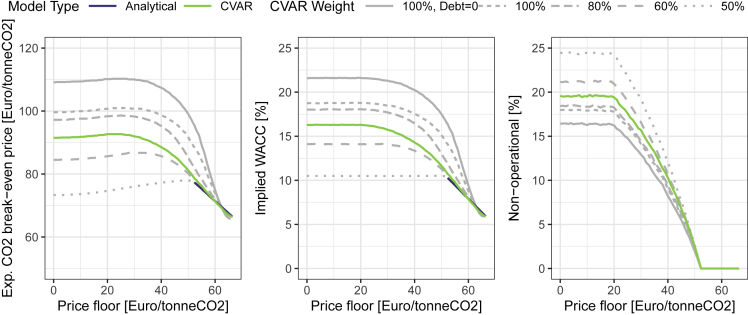
Expected carbon break-even prices, implied capital costs, and share of non-operation We show different CVaR weights as sensitivities, including one scenario with a 100% CVaR weight as a pure equity investment (100%, Debt = 0). See also Figures S1–S8.

The resulting carbon break-even prices in expectation, as well as the implied capital costs (WACC) and share of non-operation in the stochastic scenarios, are displayed in Figure 4 (cf. Figure S1 for NPVs and operational statuses in individual stochastic realizations). The Figure also shows that the chosen central CVaR model has a good fit to the analytical model with an exogenous set rate of return requirement for equity investors (the alternative parameterization is depicted in grey, further sensitivity analyses are displayed in Figures S6–S8).

As in the previous section, the result characterizes the outcome at the right-hand side of a CCfD at the break-even level of 66 Euro/t CO_2_. Moving towards the left, the result for a price floor at a lower level is depicted – assuming each time a corresponding carbon price distribution with the expected carbon break-even price on the y-axis that makes projects financially viable at the given price floor.

As can be seen, if the secured minimum carbon price falls below the level of zero debt, then the necessary expected carbon price at which projects break even increases to around 93 Euro/tonneCO_2_. In comparison, a CCfD leads to an expected break-even price of around 66 Euro/tonneCO_2_, which is around 27% lower. Break-even prices do not increase further for even lower carbon prices floors (and even slightly decrease to an optionality effect) as the steel producers can stop operating the plant and thus limit the downside risks (real option value) while profiting from the up-sides at very high carbon prices. (right-hand panel of Figure 4). The increase of carbon break-even prices is driven by the increased risk investors face of not recuperating their investment cost and can be expressed by an implied WACC (this is a result, rather than an input to the CVaR model), which nearly triples from around 5.9% to 16.3%.

**Figure 5:**
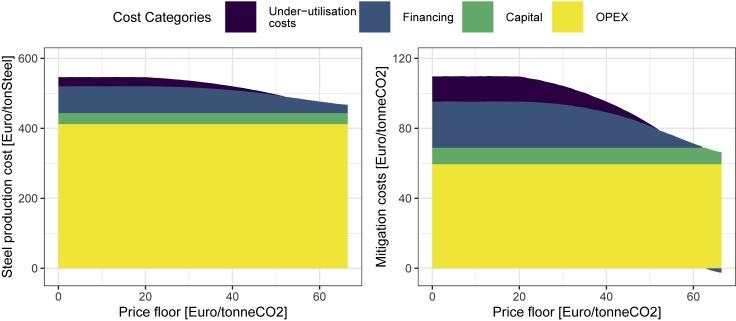
Cost categories of steel production and carbon mitigation cost The left panel shows the absolute steel production costs of the breakthrough technology, whereas the right panel shows the carbon mitigation costs (as compared to the conventional baseline). CAPEX are shown as separate subcategories (Capital, Financing, and Under-utilisation costs).

Figure 5 further differentiates the cost of steel production, and resulting carbon mitigation cost by OPEX costs and by splitting overall CAPEX in pure capital costs (at a 0% interest rate, thus without financing costs), in financing costs, as well as the implicit cost of not fully utilizing the investment in case of non-operation (under-utilization costs). Whereas the left panel shows the expected average production cost of low-carbon steel, the right panel shows the carbon mitigation costs, as compared to the dominant BF-BOF route. In the CCfD case, owing to the better financing conditions, “negative financing cost” are present in the carbon mitigation cost as compared to the conventional baseline. With falling price floors, this changes to a significant financing premium. Necessary carbon break-even prices (roughly) correspond to the sum of cost categories OPEX, capital, and financing, but not including under-utilization, as expected carbon prices include the optionality value of not running the process. The expected carbon mitigation costs are higher than the carbon prices if one accounts for the factor that (expected) non-operation results in fewer tonnes of produced steel.

## Discussion

This article analyses CCfDs as a policy tool to de-risk investments in innovative low-carbon energy-intensive industrial technologies in the context of jurisdictions with emissions trading systems, and thus potentially volatile carbon prices. We quantify the effect derisking carbon prices has on bankability, financing cost, expected break-even carbon prices, as well as carbon mitigation cost of technologies with both incremental investment costs, as well as incremental operational costs as compared to a price-setting conventional baseline. This increase in operation and investment costs broadly applies to most decarbonization technologies in energy-intensive industries (Bataille et al., 2018; IEA, 2017). To assess the impact of differing levels of carbon price risks, we compare CCfDs to more commonly discussed price floors (be it a general price floor, or a project-based put option), as well as the absence of any derisking policy. We find that CCfDs lower investment and carbon mitigation costs via three effects: they help to continuously operate the process, even at low carbon prices; they increase the amount of affordable debt the project can take on and as a result they lower financing and carbon mitigation costs.

For a case study of investments in low-carbon hydrogen steel, we find that CCfDs reduce carbon mitigation costs by around 27%, as compared to an absence of any derisking policy, and by around 14% as compared to a price floor that just enables bankability (i.e. the feasibility to take on any debt). To allow for at least some debt financing (bankability), price floors need to be relatively high (in the case study around 79% of level at which a CCfD would be issued), as otherwise the incremental operational cost and existing margins are insufficient to ensure a sufficiently secure pay-back to creditors.

For policymakers, these results indicate that if they wish to support technologies in energy-intensive industries with significant incremental operational costs and investment costs, carbon price risk (be it in emissions trading systems, or political uncertainty regarding levels of carbon taxes) is a fundamental challenge that needs to be addressed by policies. Although price floors are often touted as a universal solution to this challenge, they may fall short, when the operational carbon mitigation costs alone (partly mitigated by stable existing material price margins) are at price levels that exceed the political feasibility of general price floors, or such levels are not credible, because being perceived as regulatory instable (while contracts are legally enforceable). Project-specific contracts can be structured as contracts for difference or put options and avoid the need to find political agreement on economy-wide price floors at a sufficient scale. Put options would in principle allow for lower strike prices as investors could benefit from upsides if carbon prices exceed the strike price. However, to ensure projects can operate, also strike prices in put options would need to be set rather high and would in this case induce potential windfall profits for investors. Hence CCfDs reduce the costs to society or basic material users, as they (i) reduce financing costs and (ii) recuperate part of support from payments through revenues captured when carbon prices exceed strike prices.

We find that CCfDs are thus a useful policy complement to carbon prices for addressing the valley of death of technology upscaling, which if left unaddressed may hinder any progress in industrial emission reductions towards net-zero emissions. CCfDs do so by providing the ability to offer carbon prices above the level of market prices, and/or reducing carbon price risks, and thus financing costs. However, additional policies may be needed for efficient reduction emissions of energy-intensive material production: first, more stringent carbon pricing, including carbon leakage protection is needed to create a stable investment framework and incentivize the efficient use of materials (including material substitution). Otherwise, large-scale application of CCfDs in the absence of carbon price levels encourages inefficient use of clean materials, as they are offered below their production costs (cf. Supplemental Note S1 on how CCfDs can be made compatible with partially to fully muted carbon prices). Without basic material users ultimately paying for the incremental costs of clean production processes e.g. through carbon pricing, governments would need to dedicate scarce tax revenues to subsidize basic material production, which is neither desirable nor very credible for larger scale shift to climate neutral production processes. Second, many clean production processes require sufficient electricity supply by low-carbon sources such as wind and solar to reduce emissions, and derisking policy frameworks (such as electricity CfDs) may be needed there as well, to encourage sufficient capacity to be added at affordable prices. This is especially relevant, as electricity costs are the main cost driver for many electrified processes (cf. sensitivity in Figure S2 for the steel case study). Third, green public procurement can help in creating lead markets (Chiappinelli and Seres, 2021) and ensuring collaboration on innovation material use and production along value chains (Chiappinelli et al., 2021), but should be structured so as to ensure material-efficient design, manufacturing and construction of buildings, infrastructure and products rather than only a substitution of carbon intensive with clean produced materials funded through CCfDs. Finally, instruments to encourage a shift to climate neutral production, such as product labeling schemes to engage final consumers, financial reporting requirements to engage the finance sector, and expectations that once sufficient scale of climate neutral production is available, product carbon product requirements requiring climate neutral production processes for the sale materials in a territory will create strong long-term planning incentives to prepare for a full transition (Gerres et al., 2021; Neuhoff et al., 2019).

While the study is focused on mitigating the effect of carbon price risks in jurisdictions with emissions trading systems, CCfDs have more general relevance for decarbonizing industry, and the findings of this study may be transferred. First, while carbon taxes exhibit no explicit market risks, the underlying political uncertainty that determines the price levels over time is similar and can be hedged with CCfDs. CCfDs may also precede the introduction of carbon pricing, and start as carbon contracts, being an effective targeted subsidy for emissions reductions, that will automatically transform into a risk-hedging instrument within a general polluter-pays policy framework. By already including a clause to turn the contract into a contract-for-difference, this provides the opportunity to lower policy costs if carbon prices are eventually introduced. CCfDs may thus also be utilized as a tool of international climate diplomacy in the area of development aid, where cost-sharing clauses could additionally incentivize the introduction of carbon pricing in recipient countries (von Lüpke and Aebischer, 2021).

### Limitations of the study

The applied analytical and numerical methodologies to analyze the impact of CCfDs and price floors on financing costs of projects have several limitations. As detailed in the methodology, we assume a project finance setting, rather than a corporate finance setting. Although we argue, that owing to the basic risk-return principle, the qualitative results do not change, corporate finance may enable lower risk premiums in the absence of CCfDs. Similarly, to provide analytically tractable solutions we focus on carbon price and profit margin risks, while further risk factors, such as input factor risk differences are not the focus of the article.

We further assume that the conventional technology is price setting and that steel producers are exposed to a common varying (crude) steel price. This is on hand an abstraction of the vertical integration of the steel sector that offers heterogenous processed steel products, such as hot-rolled coil steel. On the other hand, in the long run, new processes may become dominant and price setting (in the world-wide market). Given the speed of historical technological diffusion speeds for basic steel making processes (Gold et al., 1970) this will not be relevant for the discussed timeframe.

Finally, we model the investment decision in the steel case study as a single joint investment in a full H2-DRI stack (H2 electrolyzer, direct reduction shaft, and electric arc furnace) with a single representative year of operation (such representative years are commonly used in the applied CVaR methodology and capture essential long-run uncertainties, cf. Ehrenmann and Smeers (2011)). Although we allow for operational flexibility, in the sense that the plant is not run in case operational costs are not recovered, this abstracts from several flexibilities and the optionality of the H2-DRI technology stack. First, at lower carbon prices hydrogen may be substituted with natural gas in the direct reduction shaft (if profitable), and the share of scrap as compared to sponge iron in the EAF may be increased (restricted by the desired steel quality). Both options imply that not in all cases of unfavorable conditions would the entire plant stop operation, but only parts of it (the H2 electrolyzer in the first case, and the H2 electrolyzer and DR shaft in the second). This relatively decreases the financing advantage of CCfDs as compared to put options. However, as each part of the technology stack entails significant investments, this only partly reduces, but does not absolve the associated risks, and is dependent on the availability of affordable natural gas and scrap. Second, we abstract from the short-term operational flexibility, and the ability to stockpile intermediate products, such as hot bricketed iron described by Toktarova et al. (2022) and Haendel et al. (2022). This point mainly influences the operational costs of running the process by exploiting the temporal variability of electricity prices in the short run (over periods of days and weeks) and implies a trade-off of capital and operational expenditures we ignore in our analysis. Although it is theoretically conceivable that producers opt to run the process and stockpile the output in case of low carbon prices, in contrast to current stockpiling practices such low carbon price periods may last several years and are in their lengths highly uncertain to predict, which makes this a highly risky strategy unlikely to be pursued. Further research could explore the role of further risk factors for investment decisions in clean processes as well as whether and how sufficient competition can be established in CCfD tenders, or how cost-based negotiated contracts would perform in the presence of asymmetric information and renegotiation risks.

## STAR★Methods

### Key resources table

**Table:** 

REAGENT or RESOURCE	SOURCE	IDENTIFIER
**Deposited data**		

Numeric results	Richstein and Neuhoff (2022)	https://doi.org/10.5281/zenodo.6619970

**Software and algorithms**		

Numeric CCfD & Price Floor model, as well as parameterised spreadsheet implementation of the analytical model	Richstein and Neuhoff (2022)	https://doi.org/10.5281/zenodo.6619970
R 4.1.1	R Core Team, 2021	https://www.r-project.org/
Tidyverse 1.3.1	Wickham et al. (2019)	https://www.tidyverse.org/
BB package 2019.10–01	Varadhan and Gilbert (2009)	https://CRAN.R-project.org/package=BB

### Resource availability

#### Lead contact

Please contact the lead contact, Jörn Richstein (jrichstein@diw.de) for information related to the data and code described in the following section.

#### Materials availability

This study did not generate new unique reagents.

### Method details

We investigate the impact that Carbon Contracts for Difference and carbon price floors have on financing costs and thus expected necessary carbon prices. We do so by assessing how the stabilization of carbon-price dependent revenue streams influences the financing conditions of innovative projects, most notably the debt and equity shares under project financing conditions. While in principle such projects could also be financed in a corporate finance framework, several reasons may lead project developers to choose project finance instead (Steffen, 2018). Specifically, this decision might be linked to reasons regarding the relative riskiness of the project:

1. Early investments in low-carbon technologies have risks and rewards distinctive from the core business which might lead to risk contamination (Esty, 2003) of the existing business. Without de-risking policies, the effect of “risk contamination” of the existing may lead sponsors to isolate the risk from their core business by choosing a project financing structure.
2. The decarbonization will require significant investments in new assets over a relatively short time horizon, and thus sponsors from the materials industry will face the challenge of debt overhang when financing the transformation of their business. When de-risking policies are in place (e.g. CCfDs), as in the energy sector (Egli et al., 2018; Steffen, 2018), companies may opt for using project finance.

Furthermore while financing forms do matter in practice (Steffen, 2018), the basic insights will be the same under corporate finance, as investors will still need to consider the riskiness of the investment, which even leads to identical cost of financing under idealised conditions according to the Modigliani-Miller-Theorem (Modigliani and Miller, 1958).

Thus, based on stylized project finance practices (Gatti, 2008), we develop an analytically tractable closed-form model for analyzing the financing costs of CCfDs and carbon price floors, that is valid as long as the project has sufficiently stable revenue and cost streams to be, at least in part, attractive to debt financing (“bankable”). At a higher level of risk, the model is extended by estimating equity premia using a Conditional-Value-at-Risk (CVaR) approach based on a Monte-Carlo simulation of cash flows. This risk premium under the assumption of pure equity can also be interpreted as an approximation of other financing structures (Modigliani and Miller, 1958), such as debt-equity mixes with risk premia on the debt, or the carving out of bankable subprojects (e.g. the hydrogen electrolyser). Alternative investment criteria are investigated in a sensitivity analysis.

**Table:** Parameters, variables and notation.

**Parameters**	**Explanation**
$c_{v}$	Conventional variable cost
$\text{Δ}c_{v}$	Incremental operational cost of breakthrough technology
I	Conventional overnight investment cost
ΔI	Incremental overnight investment cost of breakthrough technology
$\varepsilon_{cv}$	Emission factor conventional technology
$\varepsilon_{BT}$	Emission factor breakthrough technology
Δε	$\varepsilon_{CV}-\varepsilon_{BT}$, difference of emission factors and achieved emission reduction
$r_{D}$	Cost of debt (interest rate)
$r_{E}$	Cost of equity
$a_{D}$	Equivalent annualized percentage debt payment
$a_{E}$	Equivalent annualized percentage equity return
$d_{cv}$	Debt share for conventional technology
T	Depreciation period
$p_{G}$	Expected price of good
$p_{G,WC}$	Worst-case price of good
$p_{minCO2}$	Minimum carbon price (price floor)
**Variables**	**Explanation**
$p_{CO2}$	Necessary expected carbon price for investment
$d_{BT}$	Debt share for breakthrough technology (without CCfD)
$p_{CCfD}$	Necessary CCfD price for investment
$d_{CCfD}$	Debt share for breakthrough technology (with CCfD)

#### Analytical model for bankable projects

In the following, the closed-form financing model is described. Similarly, more applied models based on similar principles are for example used in the offshore wind industry (Mora et al., 2019). To determine the expected necessary carbon prices, we assume that a newly built conventional plant is on average price setting for a product, determining an average product price scenario. By doing so we abstract from current investment cycles in the materials sectors and focus on the long-run cost differences between technologies. Nonetheless, we incorporate the uncertainty these sectors face by estimating a worst-case product price scenario from typical financing costs and debt shares for these sectors to determine the lowest price level at which a conventional investment in a new plant can still serve its debt.

To arrive at a tractable closed-form solution, we solve the investment in an annualized framework, using debt ($a_{d}$) and equity ($a_{e}$) servicing ratios, that account both for the amortization over the project lifetime T, as well the cost of debt ($r_{d}$) and cost of equity ($r_{e}$):

(Equation 1) $$\begin{array}{l} a_{D}=\frac{r_{D}{\left(1+r_{D}\right)}^{T}}{{\left(1+r_{D}\right)}^{T}-1},\;a_{E}=\frac{r_{E}{\left(1+r_{E}\right)}^{T}}{{\left(1+r_{E}\right)}^{T}-1}\; \end{array}$$

As the interest rate for debt is usually lower than return for investment (potential explanations are adverse selection, the use of the interest rate as a screening device and resulting credit rationing (Stiglitz and Weiss, 1981)), it results in $a_{D}<a_{E}$. We assume that cost of debt and cost of equity are constant, and that their relative shares determine the overall cost of capital. While this is a simplification of reality, it is a reasonable assumption, as the relative risk both parties are taking is constant within the boundaries of the model, while the overall risk level is represented by the debt-equity share (the higher the debt level, the lower the overall risk, and resulting WACC).

In absence of a carbon price, the expected price of a good is determined by the cost of the baseline technology (in the long-run equilibrium investment and operational cost should be covered):

(Equation 2) $$\begin{array}{l} p_{G}=c_{v}+I\left(d_{CV}\cdot a_{D}+\left(1-d_{CV}\right)a_{E}\right)\; \end{array}$$

When the impact of carbon prices is considered, the assumed average equilibrium market price is incorporating the carbon emission cost, based on the emission intensity of the conventional technology (alternatively $\varepsilon_{CV}\cdot p_{CO2}\;$ can be a separate revenue stream from selling freely allocated allowances):

(Equation 3) $$\begin{array}{l} p_{G+CO2=}p_{G}+\varepsilon_{CV}\cdot p_{CO2}\; \end{array}$$

The worst-case good price is determined by the debt serving capability of the conventional technology:

(Equation 4) $$\begin{array}{l} p_{G,WC}=c_{v}+I\cdot d_{cv}\cdot a_{D}\; \end{array}$$

In other words, the worst-case good price marks the product price point where conventional producers would need to declare bankruptcy, as revenues would be insufficient to serve existing loans, and thus serves as a reasonable lower bound for good prices.

For investments in the breakthrough technology (without a CCfD), three conditions need to be fulfilled, so that an investment including debt provision takes place.

1) The investment needs to be profitable in expectation under the respective debt-equity ratios, i.e. the expected income from selling the good, including the carbon revenues, needs to be higher than the operational (including covering for carbon emission costs) and annualised investment cost:

(Equation 5) $$\begin{array}{l} p_{G}+\varepsilon_{CV}\cdot p_{CO2}\geq c_{v}+\text{Δ}c_{v}+\varepsilon_{BT}\cdot p_{CO2}+\left(I+\text{Δ}I\right)\left(d_{BT}\cdot a_{D}+\left(1-d_{BT}\right)a_{E}\right) \end{array}$$

2) In a worst-case scenario (the combination of worst-case good price and carbon price scenario, set by the carbon price floor) operational costs ($c_{v}+\text{Δ}c_{v})$ and credit payments ($\left(I+\text{Δ}I\right)\cdot d_{BT}\cdot a_{D}\;$) need to be covered (Gatti, 2008):

(Equation 6) $$\begin{array}{l} p_{G,WC}+\varepsilon_{CV}\cdot p_{minCO2}\geq c_{v}+\text{Δ}c_{v}+\left(I+\text{Δ}I\right)\cdot d_{BT}\cdot a_{D}\; \end{array}$$

3) $d_{BT}$ should be non-negative.

(Equation 7) $$\begin{array}{l} d_{BT}\geq 0\; \end{array}$$

##### General solution for price floors

Solving for the minimum viable $p_{CO2}$ (and conversely maximum debt share $d_{BT}$) where the investment takes place:

(Equation 8) $$p_{CO2}=\frac{a_{D}\cdot a_{E}\cdot \text{Δ}I\;+\;a_{D}\cdot \text{Δ}\varepsilon \cdot p_{\min CO2}\;-\;a_{E}\cdot \text{Δ}\varepsilon \cdot p_{\min CO2}+\;a_{E}\cdot \text{Δ}c_{v}}{a_{D}\cdot \text{Δ}\varepsilon }\begin{array}{l} =\frac{a_{E}\cdot \text{Δ}I}{\text{Δ}\varepsilon }+\frac{a_{E}\text{Δ}c_{v}}{a_{D}\cdot \text{Δ}\varepsilon }-\frac{a_{E}-a_{D}}{a_{D}}p_{\min CO2}\; \end{array}$$

(Equation 9) $$\begin{array}{l} d_{BT}=\frac{I\cdot a_{D}\cdot d_{CV}\;+\;\text{Δ}\varepsilon \cdot p_{minCO2}-\;\text{Δ}c_{v}}{a_{D}\left(I\;+\;\text{Δ}I\right)}\; \end{array}$$

Notably, no assumption regarding the distribution of carbon prices or good prices, except for the mean and the lower end of the distributions (relevant for determining the secure revenue streams and thus maximum debt shares) are necessary to solve the equations. Within the boundaries of the model, the necessary expected carbon price thus increases with incremental investment and operational costs and decreases with a rising carbon price floor.

##### Determining CCfD strike price and debt level

In case the project is allocated a CCfD the expected carbon price ($p_{CO2}$) and the minimum carbon price ($p_{minCO2}\;)$ are the same, and the necessary CCfD contract price and debt shares can be determined:

(Equation 10) $$\begin{array}{l} p_{CCfD}=p_{minCO2}=\frac{a_{D}\cdot \text{Δ}I\;+\text{Δ}c_{v}}{\text{Δ}\varepsilon }\; \end{array}$$

(Equation 11) $$\begin{array}{l} d_{CCfD}=\frac{I\cdot d_{CV}\;+\text{ΔI}}{I\;+\;\text{Δ}I}\; \end{array}$$

The CCfD price is thus the sum of incremental investment cost, annualised purely with the debt servicing ratio (i.e. the incremental investment cost is purely financed by a relative debt increase as compared to a conventional investment), and the incremental operational cost, divided by the achieved emissions reduction.

#### Determination of minimum bankable price floor

To derive further insight, and to define the boundary case of the model, we investigate the case, where bankability is just marginally given, and thus the debt the project can take on is approaching zero so that we can solve assuming ${\text{d}}_{\text{BT}}=0$:

(Equation 12) $$\begin{array}{l} p_{CO2,\;no\;debt}=\frac{I\cdot d_{CV}\cdot \left(a_{E}-a_{D}\right)+a_{E}\cdot \text{Δ}I+\text{Δ}c_{v}}{\text{Δ}\varepsilon }\; \end{array}$$

That is the expected carbon price, is the sum of the 1) incremental investment cost, annualised purely with the equity servicing ratio), 2) the additional cost of financing the base investment cost I via equity only, instead of at the conventional financing mix and 3) the incremental operational cost divided by the achieved emissions reductions. Notably, even if there are no incremental investment costs (or these are covered by separate public grants), the risks from volatile carbon prices contaminate the base investment cost.

(Equation 13) $$\begin{array}{l} p_{minCO2,\;no\;debt}=\frac{\Delta c_{v}-I\cdot a_{D}\cdot d_{CV}}{\Delta \varepsilon }\; \end{array}$$

The minimum price floor that enables this investment is given by the incremental operational investment cost, minus the minimum operation margin of the conventional producer in the worst-case good price scenario (given by the loan-serving capability of the conventional financing, i.e. it marks the good price point where conventional producers would need to declare bankruptcy, as revenues would be insufficient to serve existing loans, and thus serves as a reasonable lower bound for the good price expectation).

#### Numerical CVaR model

**Table:** Additional parameters and variables for Monte-Carlo Simulation

**Parameters**	**Explanation**
α	CVaR Parameter (percent worst scenarios considered)
β	CVaR Parameter (relative weight of CVaR to expected NPV)
$r_{f}$	Risk-free rate
$a_{rf}$	Equivalent annualized percentage risk free payment (via Equation 1 with $r_{f}$)
$P_{G}$	Uniform distribution of good prices ($p_{G}=E\left[P_{G}\right]$))
**Variables**	**Explanation**
$CVaR_{\alpha }$	Conditional-Value-At-Risk
E[Ν]	Expected NPV
$IC_{CVaR}$	Investment criteria – weighted average of CVaR and E[NPV]
$P_{CO2}$	Uniform distribution of carbon prices ($p_{CO2}=E\left[P_{CO2}\right]$)
N	NPV distribution

In cases of high incremental operational expenditures of the breakthrough technology, and a low minimum carbon price, as well as low worst-case product price, revenues are not sufficient to cover operational costs, and thus also any level of debt. In this case, the project is unbankable under project finance as operational costs and credit payments are not sufficiently securely covered. In this case, additional risk cannot be borne by a reduction of debt in the debt-equity share, but instead leads to an increase in the cost of equity. We take a numeric Monte-Carlo approach to iteratively find the equilibrium carbon price at which the additional return equals the additional risk and the increase in the cost of equity.

To estimate the cost of equity for investment in the break-through technology under different levels of risk we use a Conditional-Value-at-Risk (CVaR) model. We continue to represent debt, as in the analytic model. The CVaR is a coherent risk function (Artzner et al., 1999) and has been widely applied to represent risk aversion in economic models, among others in the power sector (Ehrenmann and Smeers, 2011). The CVaR limits the assessment of profitability to the worst α percent realisation. We use a weighted average of the CVaR and the expected NPV (both based on stochastic NPV realisations, discounted with the risk-free rate, as risk is endogenously represented in the model, following Ehrenmann and Smeers (2011)), as an investment criterion, that needs to be greater zero to have a positive investment decision:

(Equation 14) $$\begin{array}{l} IC_{CVaR}=\beta \cdot CVaR_{\alpha }\left[N\right]+\left(1-\beta \right)\cdot E\left[N\right]\; \end{array}$$

In the basis scenario, we calibrate the CVaR model to the conventional sector’s equity cost (cf. the section on parameterisation). We do so by assuming α to be 25% and calibrate the weighting factor β to the equity cost. Alternative parameterisations are investigated in the sensitivity analysis (cf. Figures 4 and S8).

As in the CVaR model not only the mean, and worst-case scenario are relevant (as in the analytic model), assumptions regarding the entire distribution of profit-relevant parameters and variables are needed. For simplicity, we assume that carbon prices (represented by the stochastic variable $P_{CO2}$) are distributed uniformly between 0 and $p_{CO2,max}$. In the presence of a price floor $p_{minCO2}$, the price floor is considered binding for prices below, so that it is binding in $p_{minCO2}/p_{CO2,max}$ of cases. The expected carbon price after the application of the price floor is $p_{CO2}$ and the distribution can be thus be defined by $p_{CO2}$, which is an endogenous result of the model, while $p_{minCO2}$ is an exogenous parameter. Good prices (stochastic variable: $P_{G}$) are assumed to be uniformly distributed between $p_{g,WC}$ and $p_{g,max}$, so that the expected price is $p_{g}$. The NPV distribution (N) is computed by:

(Equation 15) $$\begin{array}{l} N=max\left(P_{G}+\Delta \varepsilon \cdot P_{CO2}-c_{v}-\Delta c_{v}-a_{d}\cdot d_{BT}\cdot \left(I+\Delta I\right),0\right)-a_{rf}\cdot \left(1-d_{BT}\right)\cdot \left(I+\Delta I\right)\; \end{array}$$

As before the debt share is computed using Equation 9, with a minimum debt of 0.

Using a non-linear solver (Varadhan and Gilbert, 2009), we iterate for $p_{CO2}$ so that the investment criterion IC_{CVaR} just equals zero:

(Equation 16) $$\begin{array}{l} IC_{CVaR}\left(p_{CO2}\right)=0 \end{array}$$

As both the expected profit, as well as the CVaR depend on the carbon price distribution, which in turn depends on the variable $p_{CO2}$, for each iteration of $p_{CO2}$ a Monte-Carlo simulation is performed, where for all stochastic realisations of the simulation (i.e. the combination of realisation of the stochastic variables $P_{G}$ and $P_{CO2}$) the NPV distribution N is computed. Based on this set of numeric stochastic realisations the cost of equity and returns on equity in the carbon price scenarios can be computed.

#### Parametrization of models

The models are parameterized for the case of a commercial scale hydrogen steelmaking plant via the direct reduction route (H-DR) in Europe for the timeframe of 2025 to 2030, using recent publications regarding the techno-economic costs of this low-carbon technology and the conventional process (Vogl et al., 2018) and general steel sector financing costs (Damodaran, 2020).

Today’s dominant technology in primary steelmaking is the blast furnace – basic oxygen furnace route (BF-BOF), which uses coking coal as the primary input factor (both as a reducing agent and to deliver heat), and thus has high associated carbon emissions. Hydrogen direct reduction (H-DR) is a low-carbon technology that already has a high technological readiness level, as it builds on a conventional process that uses natural gas instead of hydrogen. The result of DR processes is direct reduced iron (DRI), which can be further processed to steel in an electric arc furnace (EAF). While several low carbon-alternative exist at different stages of technological development (Fischedick et al., 2014), incl. carbon capture and storage (CCS) technologies, the hydrogen steel making route is currently being implemented in the majority of pilot projects (Vogl et al., 2018), and can thus be considered the leading technology in low-carbon steelmaking technologies.

##### Technology cost & carbon mitigation

For the conventional BF-BOF route, we assume costs of 304 Euro/tonne of Steel as variable costs (cf. Table S1 for a disaggregation of costs in categories), as well as 442 Euro/tonne of steel production capacity as a greenfield overnight investment, following common assumptions (Vogl et al., 2018; Wörtler et al., 2013). For the new route, we assume the same operational costs as derived by Vogl et al. (2018), with base case electricity costs for the production of hydrogen of 50 Euro/MWh (corresponding to around 2.9 Euro/kg hydrogen for the conventional financing mix, and 2.8 Euro/kg in the CCfD financing mix). This results in total operational costs of the new process of 411.99 Euro/tonne steel (cf. Table S2 for disaggregation of costs). Such an electricity price is aligned with frequent exemptions for energy-intensive producers from grid tariffs and further charges world-wide (Grave et al., 2014), and current and projected costs of renewable energy systems (Bogdanov et al., 2019; Tröndle et al., 2020). For the capital costs, we assume that the DR shaft (230 Euro/tonne steel capacity) and EAF (184 Euro/tonne steel capacity) have the same overnight investment cost as the established conventional natural gas-based process (Vogl et al., 2018; Wörtler et al., 2013). We consider the electrolyser for hydrogen production to be part of the investment decision and assume a cost of 800 Euro/kW for the period of 2025–2030 (which is slightly higher than assumed by Vogl et al. (2018)). Using the same assumptions of baseload operation of the electrolyser, as well as energy flows as Vogl. et al. (2018), we arrive at an electrolyser investment cost of 216.9 Euro/tonne of steel capacity. In total, this results in an investment cost of 630.9 Euro/tonne of steel capacity, and thus 208.9 Euro/tonne of steel capacity incremental investment costs over the dominant conventional technology. Cost and energy interdependencies with up- or downstream production processes are not considered in this analysis. Following Vogl. et al. (2018), we assume that the conventional process emits 1.87 tonneCO_2_/tonne steel, and the new process emits 0.053 tonneCO_2_/tonne steel.

##### Financing cost

For the closed-form analytical model, the only parameterisations necessary are the cost of equity, the cost of debt, as well as the debt-equity share in the steel sector. For the CVaR model, these are the risk-free rate. These we directly take from the database by Damodaran (2020), who estimates these values, based on 55 stocks from the steel industry in Europe, based on the CAPM methodology. We use this as a baseline to assess the additional risks an innovative project faces, due to carbon price risks. This implicitly assumes that the historic correlation of the steel sector with the overall market (economy) remains unchanged for the future decarbonised sector. As wider parts of the economy will be impacted by carbon pricing, any estimation of the future correlation of low-carbon assets with the general market is highly uncertain, hence we assume this to be a reasonable assumption. While Damodaran suggests to correct for expected inflation (he suggests expected inflation in the US of 1.5% as compared to 0.2% in the Euro zone), recent years (2017–2019) have seen an uptick in inflation in Europe to 1.5%. Hence, no adjustment was performed.

The following table summarises the parameterization of the model for the steel sector, including references.

**Table:** Summary of model parameterisation

**Parameters**	**Value**	**Unit**	**Reference**
$c_{v}$	304	Euro/tonne steel	Vogl et al. (2018)
$\text{Δ}c_{v}$	109.58	Euro/tonne steel	Based on Vogl et al. (2018)
I	422	Euro/tonne steel capacity	Vogl et al. (2018)
ΔI	208.9	Euro/tonne steel capacity	Based on Vogl et al. (2018)
$\varepsilon_{cv}$	1.87	tonneCO_2_/tonne steel	Vogl et al. (2018)
$\varepsilon_{BT}$	0.053	tonneCO_2_/tonne steel	Vogl et al. (2018)
Δε	1.817	tonneCO_2_/tonne steel	Derived
$r_{D}$	3.06	%	Damodaran (2020)
$r_{E}$	10.27	%	Damodaran (2020)
$a_{D}$	6.76	%	Derived with Equation 1
$a_{E}$	11.97	%	Derived with Equation 1
$d_{cv}$	43.33	%	Damodaran (2020)
$p_{G}$	346.92	Euro/tonneSteel	Derived with Equation 2
$p_{G,WC}$	316.94	Euro/tonneSteel	Derived with Equation 4
$r_{f}$	1.92	%	Damodaran (2020)
α	25	%	Assumption varied in sensitivity
β		Calibrated to the risk of conventional process	Assumption varied in sensitivity

##### Validation of financing assumptions

We validate the model by comparing predicted operating margins of the conventional steel process with observed operating margins of major (crude) steel producers in Europe (the analysis script is included in the published code), as shown in Figure 1. As a basis we use EBITDA (Earnings before interest, taxes, depreciation and amortization) and sales data from Bloomberg for all major crude steel producers active in Europe for which such data was available (Voestalpine, SSAB, ThyssenKrupp, Salzgitter and ArcelorMittal). As the EBITDA data includes part of the value chain after the crude steel production, the absolute value cannot be directly used to arrive at tonne per crude steel margins. Instead we multiply the EBITDA-margin (EBITDA divided by total sales) with a crude steel price index. As crude steel is not a liquidly traded product, we develop a crude steel index based on a bottom up model of the conventional steel production process (cf. Table S1), varying the most important cost factors over time with historical data (coaking coal, steam coal and iron ore).

##### Sensitivity analyses

We perform several sensitivity analyses, both on the technological assumptions, as well as on the financial modelling (cf. Figures S2–S8). For the technological assumptions, we vary:

- The electricity cost, and thus the incremental operational cost $\text{Δ}c_{v}$ (Figure S2), as this is the major source of cost differences between the conventional and novel technologies. We alternatively assume 30 Euro/MWh and 70 Euro/MWh, resulting in incremental operational costs of 39.98 Euro/tonne steel and 179.18 Euro/tonne steel respectively.
- The baseline investment cost I in a brownfield investment scenario (Figure S3), as the question is, whether brownfield or greenfield steel investments set the equilibrium price of steel in the market. As primary steel production may peak, with increased use of recycled steel and material efficiency, refurbished, rather than greenfield conventional steel plants may be price setting (ΔI needs to be implicitly adjusted as well). We assume 170 Euro/tonne steel capacity for a brownfield retrofit (Wörtler et al., 2013).
- The incremental investment cost ΔI (Figure S4), as the incremental costs may be higher or lower than anticipated due to technological adjustments necessary for hydrogen steelmaking, as well as the electrolyser cost. We investigate an increase/decrease of 20% for the innovative investment cost (and resulting ΔI)
- The avoided carbon emissions Δε (Figure S5), as other baseline carbon emissions $\varepsilon_{CV}$ of 1.614 tonneCO_2_/tonneSteel have been estimated in literature (Otto et al., 2017), and alternative technology options for H-DRI exist, which result in remaining emissions of 0.15 tonneCO_2_/tonneSteel.

Regarding the financial modelling assumptions, we do three sensitivity analyses:

– Variation of carbon price distribution (Figure S6): The uniform distribution is in the base case assumed to start from 0 (the lowest expected carbon price, before the application of a price floor), i.e. the investors and banks see a relevant risk of the carbon price dropping to zero, or the abolition of carbon pricing. We vary this assumption by assuming higher lowest expected carbon prices (10, 20 and 30 Euro/tonneCO_2_). This has an impact on the results, even when the price floor is above the lowest expected carbon price, as this narrows the carbon price distribution.
– Sensitivity of CVaR parameters (Figure S7): we vary the relative weight of CVar as compared to the risk-neutral expected value (β); we then calibrate the worst percentage of scenarios considered (α) to match the investment decision of the conventional producer (cf. Table S3). Additionally, we investigate one case without any debt, as an abstraction from debt-equity finance is a common practice in papers, relying on the CVar approach (Ehrenmann and Smeers, 2011).
– Sensitivity of risk aversion modelling approach (Figure S8): We implement an alternative decision algorithm. Rather than using the CVaR model, we change the investment criteria, so that the average of internal rate of returns for the Monte-Carlo scenarios needs to correspond to the expected internal rate of return at the level of zero debt. Similarly, to the CVaR model, this introduces a level of risk aversion, as the IRR function is non-linear.

## Data Availability

• Model-based output data are available under an open-source license (MIT) on Zenodo. DOIs are listed in the key resource table. Figure 1 is based on data data only available under non-public licenses and is available from the lead contact upon request. • All original code has been deposited at Zenodo and is publicly available as of the date of publication. DOIs are listed in the key resources table. • Any additional information required to reanalyze the data reported in this paper is available from the lead contact upon request.

## Supplemental information

Document S1. Figures S1–S8, Tables S1–S3 and Note S1
