# Supplementary material for: Carbon contracts-for-difference: How to de-risk innovative investments for a low-carbon industry?
Source: iScience. 2022 Jul 1;25(8):104700. doi: 10.1016/j.isci.2022.104700 (PMC9287801; doi:10.1016/j.isci.2022.104700)
Supplement: Document S1. Figures S1–S8, Tables S1–S3 and Note S1 [file mmc1.pdf]

iScience, Volume *25*

Supplemental information

Carbon contracts-for-difference: How to de-risk innovative investments for a low-carbon industry?

Jörn C. Richstein and Karsten Neuhoff

Supplemental Information

Supplemental Figures

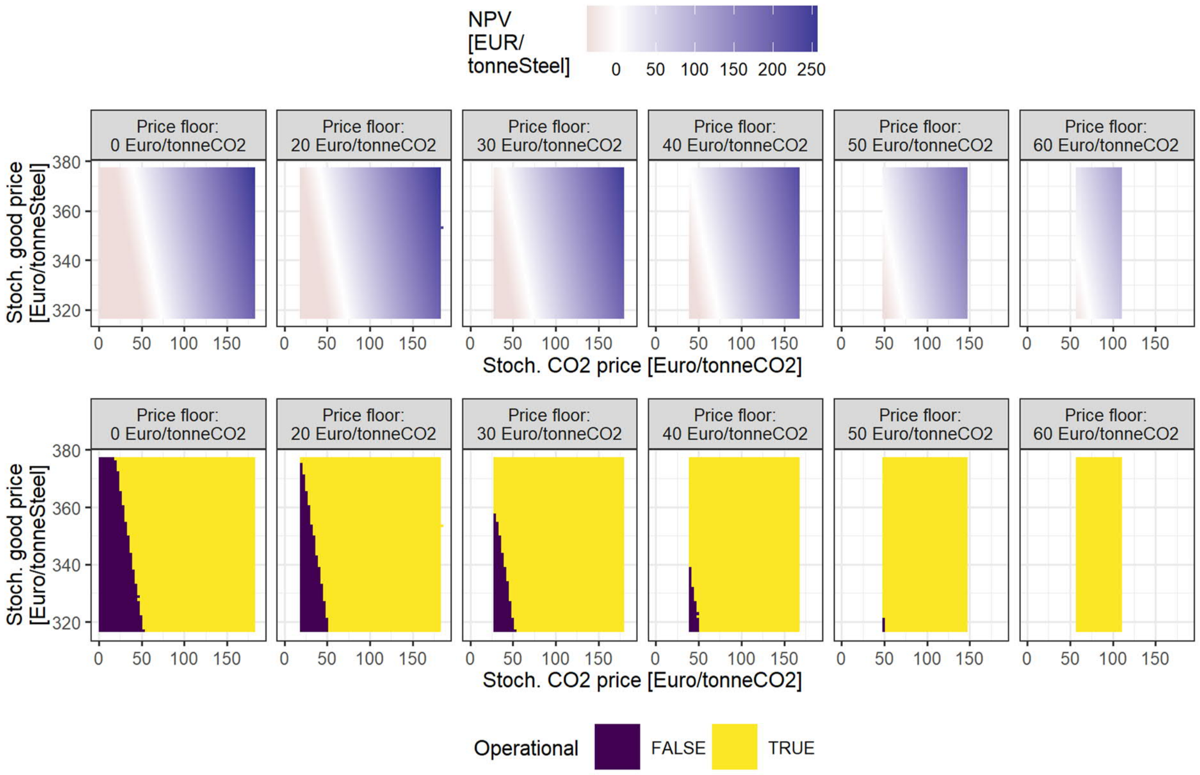

Figure S1 Net-present values at the risk-free rate and operational status at different stochastic steel and carbon price realisations in the Monte-Carlo simulation of the central scenario, related to Figure 4. Scenarios at the carbon price floor in this figure are more probable, as the price floor is binding for all carbon price realisation between zero the price floor.

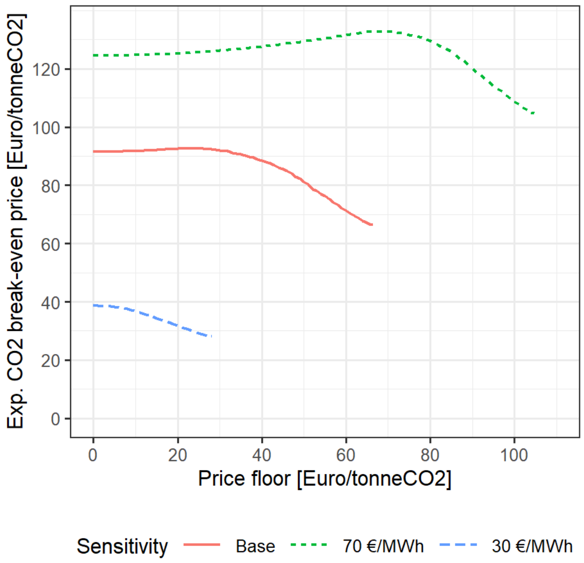

Figure S2 Sensitivity of variable cost delta via varying electricity prices (base: 50 Euro/MWh), related to Figure 4 and STAR methods

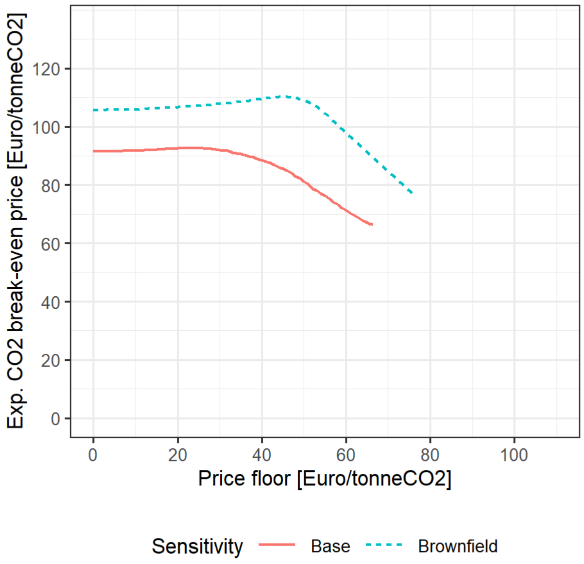

Figure S3 Sensitivity in the case of price-setting conventional brownfield technology, related to Figure 4 and STAR methods

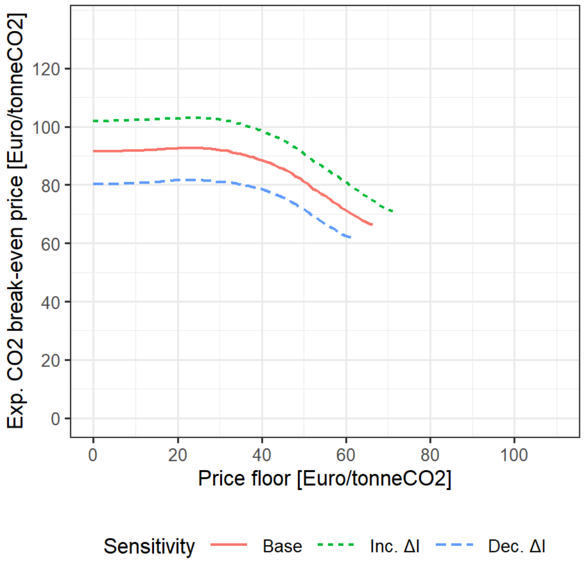

Figure S4 Sensitivity of investment cost delta (ΔI± 20%), related to Figure 4 and STAR methods

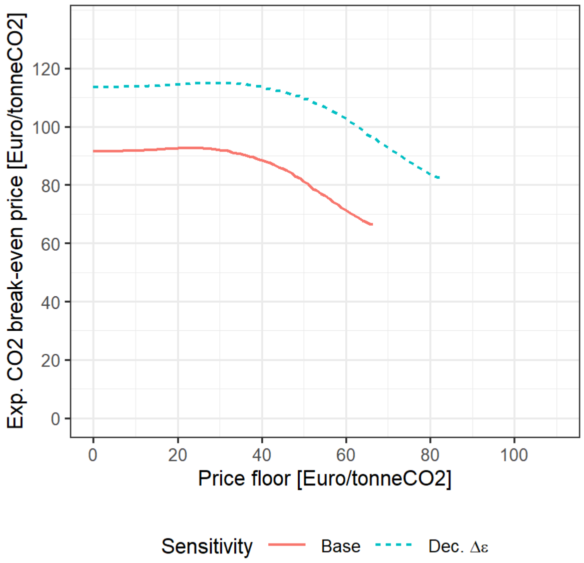

Figure S5 Sensitivity of decreased carbon mitigation (Dec. Δε: 1.46, base 1.82 tonneCO_2/tonneSteel), related to Figure 4 and STAR methods

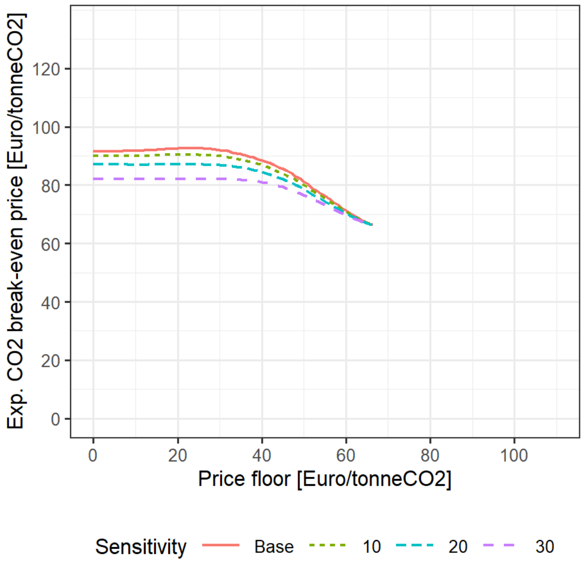

Figure S6 Sensitivity of alternative lower bound of ex-ante price floor carbon price distribution (in Euro/tonneCO2), related to Figure 4 and STAR methods

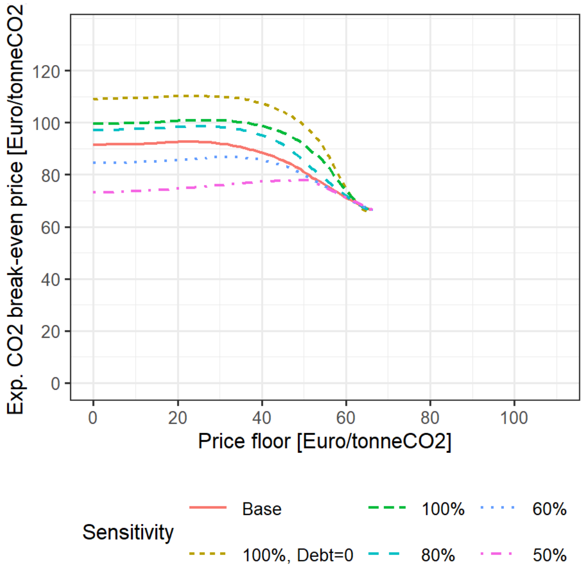

Figure S7 Sensitivity of different CVaR calibrations (Base: α=25%, calibrated β=65.7%), related to Figure 4 and STAR methods

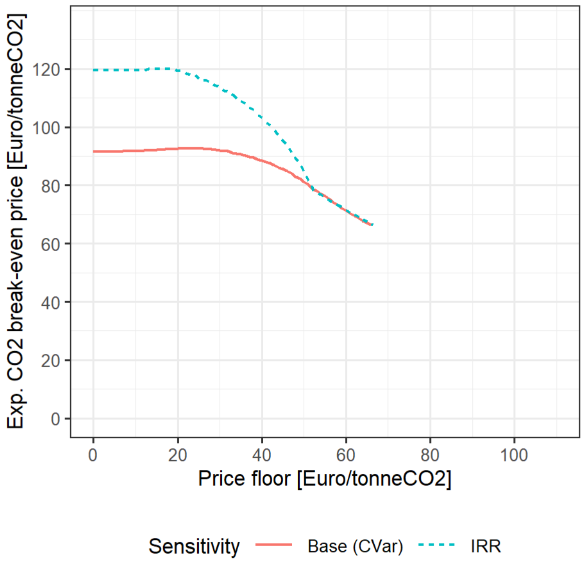

Figure S8 Sensitivity of alternative investment algorithm (assuming a constant average of IRR over all stochastic realisations), related to Figure 4 and STAR methods

Supplemental Tables

	Price	Unit Price	Input	Unit Input	Cost	References Volume	Reference price
Iron ore	*65.7*	*Euro/tonne*	*1.41*	*t/tonneSteel*	*92.64*	*Sprecher et al.*[1]	*Nasdaq*[2]
Steel scrap	180	Euro/tonne	0.21	t/tonneSteel	37.8	Sprecher et al.[1]	Vogl et al.[3]
Coking Coal	*95.60*	*Euro/tonne*	*0.55*	*t/tonneSteel*	*55.28*	*UBA*[4]	*BP*[5]
Coal dust	*53.88*	*Euro/tonne*	*0.13*	*t/tonneSteel*	*7.00*	*Sprecher et al.*[1]	*BP*[5]
Alloys	1.78	Euro/kg	11	kg/tonneSteel	19.54	Vogl et al.[3]	Vogl et al.[3]
Industrial gases (O2)	60.8	Euro/tonne	0.18	t/tonneSteel	10.94	Weigel[6]	Vogl et al.[3]
Flux	90	Euro/tonne	0.29	t/tonneSteel	29	UBA[4]	Vogl et al.[3]
Labour	53.2	Euro	1	Euro/tonneSteel	53.2	Vogl[3]	Vogl et al.[3]
O&M	3	%			1.29		
**Total**					**304**		

Table S1 Operational cost structure of conventional process (varied components in validation of financing assumption are marked italic), related to STAR methods

Type	Price	Unit Price	Input	Unit Input	Cost	Unit
Iron ore pellets	100	Euro/tonne	1.50	t/tonneSteel	150.40	Euro/tonneSteel
Scrap	180	Euro/tonne	0	t/tonneSteel	0.00	Euro/tonneSteel
Alloys	1.78	Euro/kg	11	kg/tonneSteel	19.55	Euro/tonneSteel
Flux	100	Euro/kg	0.05	kg/tonneSteel	5.00	Euro/tonneSteel
Electricity	50	Euro/MWh	3.48	MWh/tonneSteel	174.00	Euro/tonneSteel
Labour	53.2	Euro/tonneSteel	1	tonneSteel/tonneSteel	53.20	Euro/tonneSteel
Graphite electrodes	4	Euro/kg	2	kg/tonneSteel	8.00	Euro/tonneSteel
Oxygen sales	0	Euro/tonne	0.411	t/tonneSteel	0.00	Euro/tonneSteel
O&M	3	% of CAPEX			1.84	Euro/tonneSteel
**Total**					**411.99**	**Euro/tonneSteel**

Table S2 Operational cost structure of the H2-DRI process, related to STAR methods. The operational cost structure is derived from Vogel (2018), but alternative assumptions were made regarding the cost of electricity, as well as for net oxygen sales from the hydrogen, which we assume more conservatively

to be zero. The O&M cost were statically determined using the conventional debt-equity mix (debt share of 43.30%).

β (fixed)	α (calibrated)
100%, with 0 Debt	46.3%
100%	50.7%
80%	38.4%
60%	17.9
50%	1.4%

Table S3 Sensitivity Values of CVaR parameters in sensitivity analysis, related to Figure S8

Supplemental Note S1 - CCfDs and the realities of carbon pricing & leakage protection in emissions markets, related to Discussion

As materials are commodities that are heavily traded and compete on a worldwide market, they suffer from carbon leakage risks, when their production is made more expensive by carbon prices; be it taxes or emissions trading systems. Thus, producers of commodities usually do not have the market power to pass on significant shares of carbon costs to commodity prices, "muting" the carbon prices in commodity prices.

As a result, carbon pricing systems have carbon leakage protection rules, which in the case of emissions trading systems are usually implemented via free allocation for sectors deemed at risk of carbon leakage. These rules may also differ for conventional as well as novel innovative technologies, i.e. novel technologies may not receive the same level of free allocation as older technologies. Furthermore, carbon leakage rules may change, for example, if carbon border adjustment mechanisms are introduced.

These evolving carbon leakage rules have short and long-term implications for CCfDs. First, while the usual assumption of economic models is that carbon prices are passed on to consumers (at least partly), and novel technologies benefit (in the short-term when not price-setting) from the full carbon cost of the price-setting conventional technology in the revenues, while having lower costs themselves. With muted carbon prices, but full free allocation (as compared to the price-setting technology) only the source of the revenue streams changes; instead of benefiting indirectly from higher material prices, low-carbon producers benefit from the sale of (unused) free allowances. As Vogl et al.[7] and Sutherland[8] highlight CCfDs are compatible with both policy regimes.

For other cases, such as partial free allocation, a globally agreed (minimum) carbon price for material producers or a partly introduced CBAM, the concept of "effective" carbon prices can be introduced. This is defined via carbon prices that are indeed revenue-effective (carbon revenues R_{CO2}, e.g. from pass-through of carbon prices in material prices, or sales of free allowances) and cost-effective for producers (carbon costs C_{CO2}, e.g. costs under a carbon pricing system for remaining emissions of the technology).

$$p_{CO2,effective} = \frac{R_{CO2} - C_{CO2}}{\Delta\varepsilon}$$

CCfDs can then be used to (temporally) resolve muted carbon prices due to global competition, or carbon leakage rules. Importantly this definition also extends to the border case of a total absence of carbon pricing, where the effective carbon price would be zero (but may be adjusted over time, if carbon pricing is introduced); as well as to the other border case where the effective carbon price is equal to emissions market price, or carbon tax ($p_{CO2,effective} = p_{CO2}$), in the case of full carbon pass-through, or full free allowance allocation). The payment from the CCfD contract is defined as:

$$\zeta_{CCfD} = \left(p_{CCfD} - p_{CO2,effective}\right) \cdot \Delta\varepsilon \cdot q$$

If carbon prices are muted, or free allowance allocation is absent, this will lead to higher costs to governments from CCfDs. However, depending on the carbon leakage mechanism this may be recuperated elsewhere, for example via an excise tax or consumption charges[9].

Supplemental References

1. Sprecher M, Lüngen HB, Stranzinger B, Rosemann H, Adler W. Abwärmenutzungspotenziale in Anlagen integrierter Hüttenwerke der Stahlindustrie [Internet]. Stahlinstitut VDEh; 2019. (Texte). Report No.: 07/2019. Available from: https://www.umweltbundesamt.de/publikationen/abwaermenutzungspotenziale-in-anlagen-integrierter

2. Nasdaq Data Link. IMF, China import Iron Ore Fines 62% FE spot (CFR Tianjin port) [Internet]. 2022 [cited 2022 Jan 14]. Available from: https://data.nasdaq.com/data/ODA/PIORECR_USD-china-import-iron-ore-fines-62-fe-spot-cfr-tianjin-port-us-dollars-per-metric-ton

3. Vogl V, Åhman M, Nilsson LJ. Assessment of hydrogen direct reduction for fossil-free steelmaking. Journal of Cleaner Production [Internet]. 2018;203:736–45. Available from: http://www.sciencedirect.com/science/article/pii/S0959652618326301

4. UBA. Merkblatt über die Besten Verfügbare Techniken in der Eisen- und Stahlerzeugung nach der Industrie-Emissionen-Richtlinie 2010/75/EU [Internet]. 2012. Available from: https://www.umweltbundesamt.de/sites/default/files/medien/367/dokumente/bvt-merkblatt_eisen-_und_stahlerzeugung_endfassung.pdf

5. BP. BP Statistical Report [Internet]. 2021 [cited 2021 Jan 14]. Available from: https://www.bp.com/en/global/corporate/energy-economics/statistical-review-of-world-energy.html

6. Weigel M. Ganzheitliche Bewertung zukünftig verfügbarer primärer Stahlherstellungsverfahren [Internet] [Wissenschaftliche Abschlussarbeiten » Dissertation]. Universität Wuppertal, Fakultät für Maschinenbau und Sicherheitstechnik » Sicherheitstechnik » Dissertationen; 2018 [cited 2022 Jan 14]. Available from: http://elpub.bib.uni-wuppertal.de/servlets/DocumentServlet?id=4650

7. Vogl V, Åhman M, Nilsson LJ. The making of green steel in the EU: a policy evaluation for the early commercialization phase. Climate Policy [Internet]. 2021 Jan 2 [cited 2021 Dec 17];21(1):78–92. Available from: https://doi.org/10.1080/14693062.2020.1803040

8. Sutherland BR. Accelerating Green Steel in the EU. Joule [Internet]. 2020 Sep 16 [cited 2022 Mar 16];4(9):1860–1. Available from: https://www.sciencedirect.com/science/article/pii/S2542435120303998

9. Pollitt H, Neuhoff K, Lin X. The impact of implementing a consumption charge on carbon-intensive materials in Europe. Climate Policy [Internet]. 2020 Apr 22;20(sup1):S74–89. Available from: https://doi.org/10.1080/14693062.2019.1605969
